# A Patient Self-Checkup App for COVID-19: Development and Usage Pattern Analysis

**DOI:** 10.2196/19665

**Published:** 2020-11-06

**Authors:** JoonNyung Heo, MinDong Sung, Sangchul Yoon, Jinkyu Jang, Wonwoo Lee, Deokjae Han, Hyung-Jun Kim, Han-Kyeol Kim, Ji Hyuk Han, Woong Seog, Beomman Ha, Yu Rang Park

**Affiliations:** 1 Armed Forces Medical Command Seongnam Republic of Korea; 2 Department of Biomedical Systems Informatics Yonsei University College of Medicine Seoul Republic of Korea; 3 Department of Medical Humanities and Social Sciences Yonsei University College of Medicine Seoul Republic of Korea; 4 Department of Ophthalmology Yonsei University College of Medicine Seoul Republic of Korea; 5 Companoid Labs Yonsei University Seoul Republic of Korea; 6 Department of Neurology Yonsei University College of Medicine Seoul Republic of Korea; 7 Department of Internal Medicine The Armed Forces Capitol Hospital Seongnam Republic of Korea; 8 Department of Otorhinolaryngology The Armed Forces Capitol Hospital Seongnam Republic of Korea

**Keywords:** COVID-19, mobile app, smartphone, mobile phone, self-checkup

## Abstract

**Background:**

Clear guidelines for a patient with suspected COVID-19 infection are unavailable. Many countries rely on assessments through a national hotline or telecommunications, but this only adds to the burden of an already overwhelmed health care system. In this study, we developed an algorithm and a web application to help patients get screened.

**Objective:**

This study aims to aid the general public by developing a web-based application that helps patients decide when to seek medical care during a novel disease outbreak.

**Methods:**

The algorithm was developed via consultations with 6 physicians who directly screened, diagnosed, and/or treated patients with COVID-19. The algorithm mainly focused on when to test a patient in order to allocate limited resources more efficiently. The application was designed to be mobile-friendly and deployed on the web. We collected the application usage pattern data from March 1 to March 27, 2020. We evaluated the association between the usage pattern and the numbers of COVID-19 confirmed, screened, and mortality cases by access location and digital literacy by age group.

**Results:**

The algorithm used epidemiological factors, presence of fever, and other symptoms. In total, 83,460 users accessed the application 105,508 times. Despite the lack of advertisement, almost half of the users accessed the application from outside of Korea. Even though the digital literacy of the 60+ years age group is half of that of individuals in their 50s, the number of users in both groups was similar for our application.

**Conclusions:**

We developed an expert-opinion–based algorithm and web-based application for screening patients. This innovation can be helpful in circumstances where information on a novel disease is insufficient and may facilitate efficient medical resource allocation.

## Introduction

On March 11, 2020, the World Health Organization officially characterized COVID-19, a disease caused by the novel coronavirus SARS-CoV-2, as a pandemic [1]. As general preventive guidelines, hygiene control and social distancing were recommended, but in a situation where one shows signs of infection, there are no clear guidelines for patients on how to respond. Many countries, including South Korea, rely on individual assessments and advice provided by a health care worker. This could be carried out by calling a national hotline or one’s local clinic, or by means of telecommunication that assesses and advises people on what to do in the event of a suspected infection [2]. This often results in overwhelmed call centers with long waiting times and untimely response to emergency patients.

Viral pandemics such as COVID-19 can place extraordinary and sustained demands on public health systems and essential community service providers [3]. Patient triage is essential in such situations to efficiently allocate resources [4,5]. In addition, patients need to be advised on the adequate care measures to be taken not only for themselves but also for others around them. However, for novel infections like COVID-19, it is difficult for both the general public and less experienced medical staff to accurately make decisions.

Traditionally, for medical advice to be made public, many trials have to repetitively prove that the advice is sound and without any unforeseen side effects. This is, in many ways, appropriate, considering the consequences of erroneous recommendations. However, in the current situation, patients who stand a chance of survival with professional care may still die due to a lack of clinical resources. Thus, a well-timed, imperfect solution may be more beneficial than belated, flawless advice. In addition, this information can easily be confusing to the general public, and false interpretation may cause harm to users. Providing medical knowledge in a friendly and clear manner through an application can be both satisfactory and safe for users. Providing screening algorithms based on experiences from actively involved physicians can help improve the efficiency of the diagnosis process. This is made possible by both accurately selecting the patients to be tested and alleviating the workload imposed on health care workers.

In this study, we developed an algorithm and a web application for use by the general public to help them decide whether or not to seek professional care in cases where a COVID-19 infection is suspected.

## Methods

### Algorithm Development

During the initial phases of the COVID-19 outbreak in Korea, the government and the Korean Center for Disease Control and Prevention (KCDC) demonstrated expansive and extensive efforts to isolate infected people and trace and quarantine their contacts. The KCDC guidelines stated that two groups of patients be tested: suspected cases and patients under investigation [6]. A person exhibiting fever or respiratory symptoms within 14 days of contact with a confirmed COVID-19 case was defined as a suspected case. Patients under investigation were defined as those who traveled overseas or those with an epidemiological link to a domestic COVID-19 cluster with fever or respiratory symptoms. In addition, a person suspected to have COVID-19 according to a physician’s opinion was also classified as a patient under investigation and was subject to COVID-19 testing. Many patients were tested based on their physician’s opinion, and no clear guidelines existed on features to be considered.

Our algorithm was built with the aim of helping a patient decide when they should consult a doctor for COVID-19 testing. It was developed through the consultation of a group of physicians (n=6) directly involved in the process of screening, diagnosis, and/or treatment of patients with COVID-19. The KCDC guidelines were also reviewed for any information on patient selection for COVID-19 testing [6].

The physician group comprised 1 pulmonologist and 1 infectious disease specialist directly involved in the care of confirmed cases; 1 pulmonologist and 1 otorhinolaryngologist involved in patient screening at the hospital; and 2 neurologists involved in patient screening at public health centers. Based on experience and a literature review, clinical variables that should be considered when screening for patients with COVID-19 were selected, and the decision process of selecting patients to be tested for COVID-19 was discussed.

**Figure 1:**
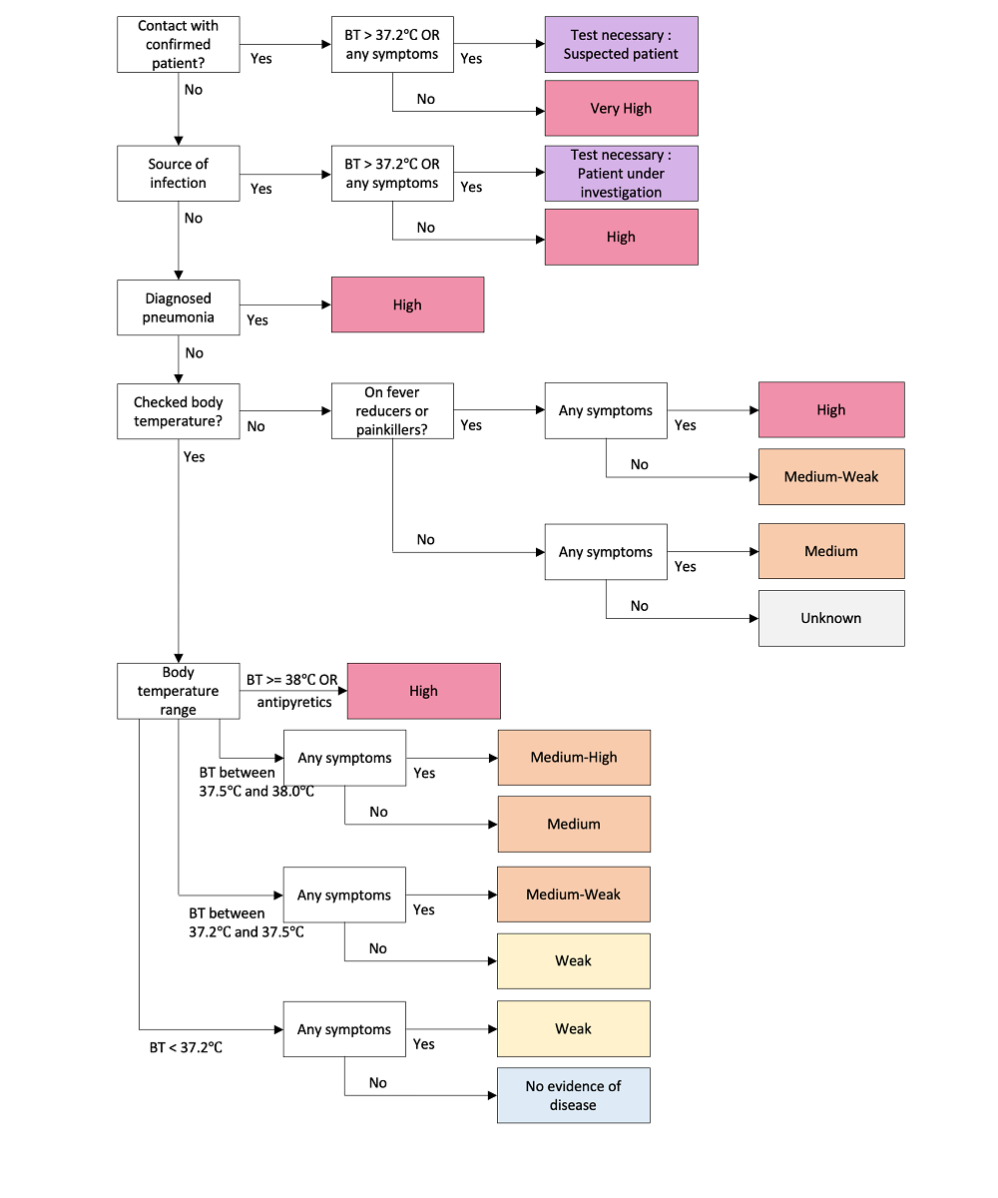
Algorithm for the COVID-19 CheckUp app. BT: body temperature.

The final algorithm of COVID-19 CheckUp is shown in Figure 1. Upon reviewing the KCDC guidelines [6] and several papers in the literature [7-9], the patients who were required to report to the KCDC and undergo a COVID-19 test were clearly defined. KCDC guidelines state that patients who show any symptoms of upper respiratory infection or documented fever combined with direct contact with an individual with confirmed COVID-19 or have visited a well-known outbreak area should report to the KCDC and take a COVID-19 confirmatory test immediately.

The first part of the algorithm was designed to meet the KCDC guidelines. The user is asked if there was any contact with a confirmed patient or any other source of infection. If the user answered “yes” to these questions and presented any relevant symptoms or fever, they are instructed to report to the KCDC and take a COVID-19 test. A user who had contact a confirmed COVID-19 case is still classified as highly likely to be needing a test even if the patient presents no symptoms and has a normal body temperature.

Body temperature was classified into four categories: high fever (higher than 38 °C), fever (higher than 37.5 °C but lower than 38 °C), mild fever (higher than 37.2 °C but lower than 37.5 °C), and normal (lower than 37.2 °C). Body temperature was chosen as the most important variable for the process of screening as it was one of the most objective variables the user could provide; further, the pathogens in patients with high fever were more likely to be viral. In addition, patients using antipyretics, including nonsteroidal anti-inflammatory drugs (NSAIDs), were treated like patients with high fever. This was done to reflect cases where fever and symptoms of upper respiratory infection were masked by the chronic use of antipyretics; additionally, patients who took these medications were usually at a higher risk because of their chronic comorbidities. The detailed classification of temperature into four categories (high fever, fever, mild fever, and normal) was used to consider the numerous reports of patients with COVID-19 presenting with only mild fever [10], and at the same time differentiate patients with high fever, who should be considered more gravely.

### Application

**Figure 2:**
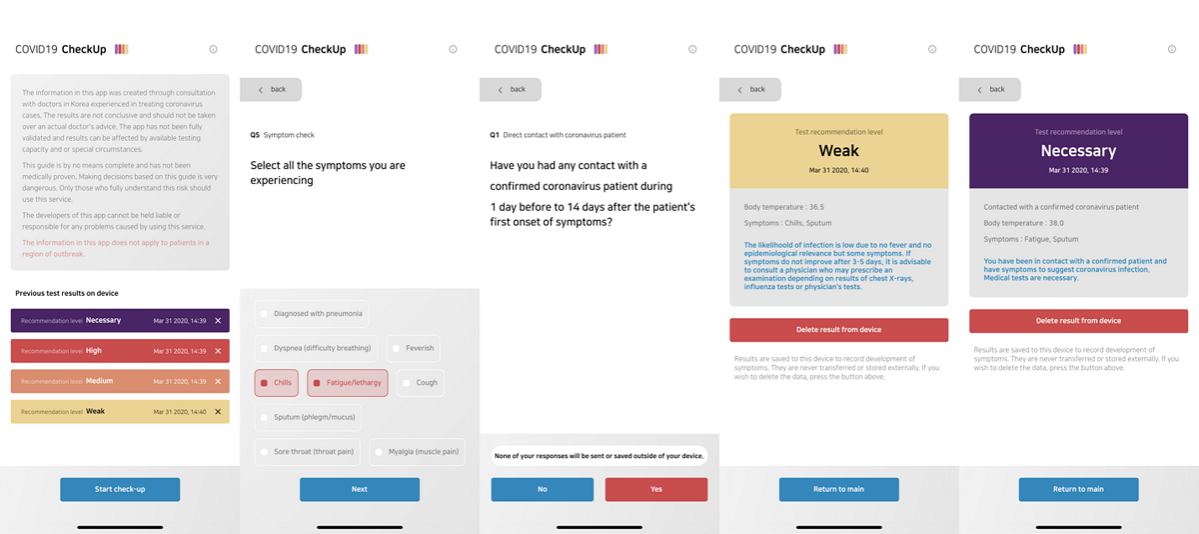
Screenshots of the COVID-19 CheckUp app.

COVID-19 CheckUp was developed using the Flutter software development kit [11]. It was deployed as a web application [12] for better accessibility and maintenance. The application has been designed to be mobile-friendly and interactive. HTML5 local storage was used to store users’ data, which ensures that usage data are never transferred to a server. This structure ensures that the privacy of users’ data can be protected and at the same time eliminates the need for a back-end architecture. To better accommodate international usage, the application has been translated into 5 languages: Korean, English, French, Spanish, and Vietnamese. Screenshots of the COVID-19 CheckUp app are shown in Figure 2. When using the app, the patient is asked a maximum of 7 simple questions based on our algorithm.

### Usage Patterns

To gain insight on application usage, Google Analytics [13] was embedded and usage data were collected from March 1 to March 27, 2020. Google Analytics API [14] was used to further help extract data from Google Analytics. The extracted data include frequency of access and location of the user. The data on COVID-19 cases and deaths were extracted from Johns Hopkins University’s COVID-19 data repository on GitHub [15]. The usage numbers were compared by subcontinent and city. Screened cases for each city were also included for analysis. Each local autonomous body in Korea released the number of COVID-19 tests that were conducted. For Indonesia, because data on the number of assessments were not available, the number of screened cases was defined as the sum of patients classified as “under monitoring,” “under supervision,” and “confirmed.” Moreover, usage by age group was compared to their corresponding digital literacy and COVID-19 fatality by age (sourced from the KCDC). Digital literacy by age was obtained from a report on the digital divide in 2019 released by the Ministry of Science and ICT and the National Information Society Agency in Korea [16].

All the statistical analyses were performed using R 3.6.0 (The R Foundation).

## Results

In total, 83,640 users accessed the application 105,508 times from 141 countries. In Eastern Asia, where Korea is located, 43,648 users accessed the application 57,030 times. Table 1 reports the number of application users by subcontinent compared with the number of COVID-19 confirmed cases and deaths. Although the application had not been introduced or advertised outside of Korea, the website received heavy traffic from other subcontinents in the following order: Southeast Asia, North America, and Western Europe (Multimedia Appendix 1).

**Table 1:** The number of users, new users, sessions, COVID-19 cases, and deaths by subcontinent.

Subcontinent	Users (n=83,640), n	Sessions (n=105,508), n	Cases (n=7,584,046), n	Deaths (n=289,731), n
Eastern Asia	43,648	57,030	4,103,263	135,335
Southeast Asia	31,117	38,033	52,604	1083
North America	5767	6653	499,451	7112
Western Europe	734	881	817,082	16,536
Australasia	575	661	21,042	125
Northern Europe	400	495	200,750	4571
Western Asia	301	331	75,591	875
South America	263	356	49,203	748
Southern Europe	251	315	1,271,012	98,153
Eastern Europe	136	164	44,315	400
Central America	83	100	9037	114
Southern Asia	78	102	384,601	23,763
Central Asia	66	84	1516	6
Northern Africa	64	77	10,085	477
Eastern Africa	53	94	1509	20
Sub-Saharan Africa	25	33	3558	73
Southern Africa	21	30	5122	1
Caribbean	20	25	4001	85
Unidentified	16	17	—^a^	—
Micronesia	15	19	—	—
Middle Africa	7	8	1073	25

^a^Not applicable.

Table 2 shows the list of the top 10 cities with the highest user ratios (user count per 100,000 population). The majority of users were from Korea (n=43,210, 51.64%); almost half (n=40,907, 48.36%) were from outside of Korea.

In Korea, the digital literacy of users in their 50s and 60s is 73.60% and 35.70%, respectively. However, application usage was similar between the two groups at 5.5% each. Even though the digital literacy of users in their 60s was half that of users in their 50s, the user count was similar for our application (Table 3).

**Table 2:** Top 10 cities with the highest user ratios.

City	Country	Users^a^, n	Population, N	User ratio^b^	Confirmed, n	Confirmed ratio^b^	Screened^c^, n	Screened ratio^b^
Seoul	South Korea	22,542	10,010,983	225.17	360	3.60	65,952	658.80
Busan	South Korea	4768	3,459,840	137.81	112	3.24	—^d^	—
Jakarta	Indonesia	13,646	10,504,100	129.91	515	4.90	3704	35.26
Depok	Indonesia	2866	2,727,209	105.09	10	0.37	634	23.25
Bandung	Indonesia	2093	2,580,191	81.12	3	0.12	221	8.57
Daegu	South Korea	1805	2,468,222	73.13	6482	262.62	—	—
Surabaya	Indonesia	2110	2,944,403	71.66	31	1.05	217	7.37
Daejeon	South Korea	921	1,493,979	61.65	31	2.07	8201	548.94
Incheon	South Korea	1293	3,029,285	42.68	47	1.55	15,219	502.40
New York	United States	534	8,398,748	6.36	20,011	238.26	—	—

^a^Users from March 1 to 27, 2020.

^b^Ratio defined as count per 100,000 people.

^c^In Korea, screened cases indicate the patients who underwent COVID-19 testing, but in Indonesia, the number of screened cases were defined as the sum of patients classified as under monitoring, under supervision, and confirmed.

^d^Not available.

**Table 3:** Application usage, digital literacy, and mortality, compared by age group, in Korea.

Variable	Age group					
	20-29 years	30-39 years	40-49 years	50-59 years	60-69 years	≥70 years
Application usage^a^, n (%)	23,001 (27.50)	28,019 (33.50)	12,964 (15.50)	10,455 (12.50)	4600 (5.50)	4600 (5.50)
Digital literacy^b^ (%)	112.30	123.00	121.70	112.70	73.60	35.70
Fatality cases, n (%)^c^	0 (0)	1 (0.10)	1 (0.08)	10 (0.56)	21 (1.75)	111 (10.43)

^a^Estimated users from age group percentage and total number of users.

^b^Digital literacy is expressed as a relative score to the average literacy of the Korean public (score=100). A group score >100 indicates that the digital literacy of that group is higher than that of the general public.

^c^The COVID-19 fatality rate (%) is defined as an occurrence of death from a confirmed case of COVID-19.

## Discussion

### Principal Findings

This study explains the development and deployment of a globally used COVID-19 symptom checkup application. The main focus of this solution was to provide information about the coronavirus disease, including which symptoms should be taken seriously and under what circumstances COVID-19 should be suspected. Many measures were taken to “flatten the curve,” but there were not enough medical resources to smoothly handle the overwhelming spread of the virus [17]. Inevitably, there was mass confusion and distress among the public, which in turn worsened the shortage of medical resources. Our application aimed to break this cycle with, however imperfect, timely knowledge of the unknown virus. This study describes the development of an application to screen for a novel emerging infectious disease that can be deployed worldwide. The usage pattern of the application was not proportional to digital literacy. The application was quickly adapted for use in the military and the government.

Medical resources may not always be sufficient, especially in a pandemic situation. Traditionally, consultation through telecommunication, such as a national hotline, was used to triage patients. This method may be more thorough than a simple decision support system. However, considering how the spread of the virus is overwhelming the entire health care industry, using ICT (information and communications technology) to alleviate the workload of health care workers can be beneficial.

Upon building the algorithm for the application, all the participating physicians easily concluded that there cannot be a decisive method to screen patients for COVID-19 infection. This is mainly because many patients are asymptomatic, with some studies reporting that up to 75% of patients can show no symptoms [18-21]. Hence, the algorithm presents a “no evidence” rather than “testing not needed” result when there is no evidence to indicate COVID-19 infection. A total of 10 levels of risk are presented to the user, ranging from “test necessary” and “very high” to “unknown” and “no evidence.” The fine division of the risk level is to inform the user of the factors that should be considered, and which factors are more important than others, for testing.

Web-based applications are easily accessible because they can be used with a typical preinstalled web browser, rather than having to install a separate app. Moreover, even though our application was developed for a mobile interface, it can be used on any devices such as desktops that connect to the internet. This wide range of supported devices may be beneficial for environments where a mobile device is unavailable.

Without any advertising abroad, the fast adoption of our application overseas reflects the need for such a solution amidst the general public. The application was translated into a total of 5 languages, increasing accessibility for global users. Referrer data acquired using the Google Analytics tool showed that 20.95% of user acquisition was from Twitter, which may account for the worldwide usage of the application. Additionally, the application was registered in the World Health Organization Digital Health Atlas [22].

There are concerns regarding ICT accessibility (eg, too complicated to use or unfamiliar to an older audience). However, our study shows that the user distribution is unexpectedly higher in the older age groups with respect to the digital literacy rate of older adults [16]. Considering that disease fatality increases among elderly patients [23], users’ needs seem to outweigh the discomfort of new technologies. The application is constructed as a series of pages with only one question per page. This simple question-and-answer type interface may be more familiar to the elderly user group. However, additional research is needed to confirm the hypothesis.

### Comparison With Prior Works

There are other applications that help users to screen themselves. Apple Inc, in association with the Centers for Disease Control and Prevention, released the web-based COVID-19 Screening Tool [24]. In addition to physicians, the app also focused on patients, and it was found to be useful in the United States. Spector and colleagues developed the COVID-19 Symptom Tracker app in the United Kingdom, with a partnership between researchers at King’s College London and the health-related data science company ZOE [25]. Judson et al [26] introduced a self-triage and a self-scheduling tool based on well-designed algorithms. Integrated into the electronic medical record (EMR), it enables physicians to easily use the application and possibly enable automatic data collection of user-provided data. EMR-tethered solutions are effective for local users but not for use outside of the region where the associated EMR system is not available. In Korea, the Self-Check Mobile App has been developed by the Ministry of Health & Welfare to monitor symptoms in people for 14 days who arrived in Korea from abroad [27]. We provide a list of similar applications in Multimedia Appendix 2.

The application described in this paper, the COVID-19 CheckUp app, is officially in use in military hospitals and throughout the military service line. Use of the app to check symptoms is recommended to all personnel with suspected COVID-19 symptoms. Moreover, the city of Seoul has offered to officially support our project by placing a banner linked to our application on their website. Further discussions with government departments are underway.

### Limitations and Conclusion

There are limitations to this study. The algorithm was based on KCDC guidelines, review of a few publications, and the expert opinions of 6 physicians. Evidence-based guidelines should be developed using data from patients with COVID-19. Insufficient data were available to develop an evidence-based model at the time, but additional studies developing statistical models using the newly acquired patient data will be followed. Additionally, one of the most important variables in the algorithm is body temperature. The typical household thermometer, especially the noncontact infrared thermometers, may be inaccurate, providing false guidance to users [28-30]. Another limitation of this study is that it does not provide any validation results on whether the algorithm was successful in patient triaging or increased efficiency in resource allocation. Concerns about the users’ privacy led to the development of an application that does not collect or send any information provided by the user. Thus, no data were available for use in validating the efficacy of the model. Further, there is regional bias in the usage pattern since the application was developed and advertised only in Korea. However, many people in other countries have used the app since it supports multiple languages. Moreover, the usage pattern was not analyzed temporally because it was difficult to access all the temporal marks that can affect usage patterns.

An expert-opinion–based algorithm and application for patient screening and guidance can be beneficial in a circumstance where there is insufficient information on a novel disease and medical resources are limited.

## Appendix

Multimedia Appendix 1 Geographical distribution of app users.

Multimedia Appendix 2 List of COVID-19–related apps.

## Abbreviations

EMR: electronic medical record
ICT: information and communications technology
KCDC: Korean Center for Disease Control and Prevention
NSAID: nonsteroidal anti-inflammatory drug
