# Supplementary figures and images for: A Patient Self-Checkup App for COVID-19: Development and Usage Pattern Analysis
Source: J Med Internet Res. 2020 Nov 6;22(11):e19665. doi: 10.2196/19665 (PMC7652594; doi:10.2196/19665)

Multimedia Appendix 1. Geographical distribution of application users.


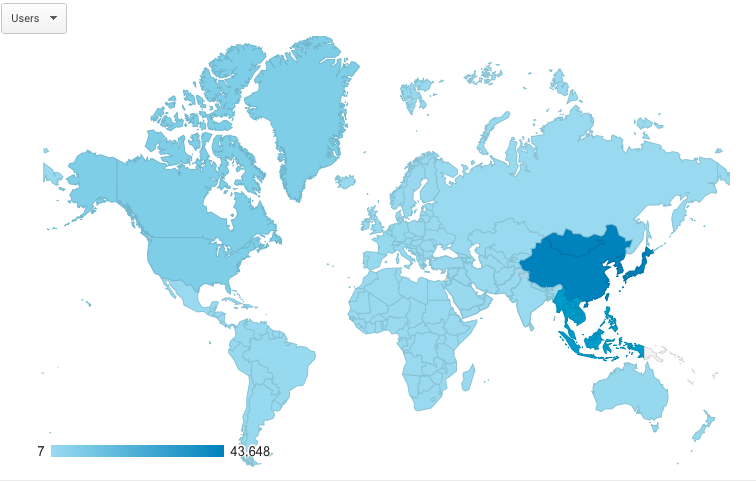

Supplement: Multimedia Appendix 1 [file jmir_v22i11e19665_app1.docx]
