# Supplementary material for: A Patient Self-Checkup App for COVID-19: Development and Usage Pattern Analysis
Source: J Med Internet Res. 2020 Nov 6;22(11):e19665. doi: 10.2196/19665 (PMC7652594; doi:10.2196/19665)
Supplement: Multimedia Appendix 2 [file jmir_v22i11e19665_app2.docx]

Multimedia Appendix 2. List of COVID-19–related apps.

|  | **Country** | **Name** | **Functionality** | **Platform** |
| --- | --- | --- | --- | --- |
| 1 | Angola | COVID-19 AO | self diagnostic, information and quarantine enforcement | Web |
| 2 | [Australia](https://en.wikipedia.org/wiki/Australia) | [Coronavirus Australia](https://en.wikipedia.org/wiki/Coronavirus_Australia) | information, isolation registration | Android, iOS |
| 3 |  | [COVIDSafe](https://en.wikipedia.org/wiki/COVIDSafe) | contact tracing | Android, iOS |
| 4 | [Austria](https://en.wikipedia.org/wiki/Austria) | [Stopp Corona](https://en.wikipedia.org/w/index.php?title=Stopp_Corona&action=edit&redlink=1) | contact tracing, medical reporting | Android, iOS |
| 5 | [Brazil](https://en.wikipedia.org/wiki/Brazil) | The Spread Project | contact tracing, medical reporting | Android, iOS |
| 6 | [Canada](https://en.wikipedia.org/wiki/Canada) | [COVID Shield](https://en.wikipedia.org/w/index.php?title=COVID_Shield&action=edit&redlink=1) | contact tracing | Android, iOS |
| 7 | [China](https://en.wikipedia.org/wiki/China) | ["Alipay Health Code"](https://en.wikipedia.org/w/index.php?title=Alipay_Health_Code&action=edit&redlink=1) | contact tracing | Android, iOS |
| 8 | [Czech Republic](https://en.wikipedia.org/wiki/Czech_Republic) | [eRouška](https://en.wikipedia.org/w/index.php?title=ERou%C5%A1ka&action=edit&redlink=1) | contact tracing | Android, iOS |
| 9 | [Finland](https://en.wikipedia.org/wiki/Finland) | Ketju | contact tracing | Android, iOS |
| 10 | [France](https://en.wikipedia.org/wiki/France) | StopCovid [fr] | contact tracing | Android, iOS |
| 11 |  | ROBERT (ROBust and privacy-presERving proximity Tracing protocol) | contact tracing | *unknown* |
| 12 | [Georgia](https://en.wikipedia.org/wiki/Georgia_(country)) | [Stop Covid](https://en.wikipedia.org/w/index.php?title=Stop_Covid&action=edit&redlink=1) | contact tracing | Android, iOS |
| 13 | [Germany](https://en.wikipedia.org/wiki/Germany) | [Ito](https://en.wikipedia.org/w/index.php?title=Ito_App&action=edit&redlink=1) | contact tracing | Android |
| 14 |  | [OHIOH Framework](https://en.wikipedia.org/w/index.php?title=OHIOH_App&action=edit&redlink=1) | contact tracing, scientific research | Android, OS |
| 15 | [Greece](https://en.wikipedia.org/wiki/Greece) | [DOCANDU Covid Checker](https://en.wikipedia.org/w/index.php?title=DOCANDU_Covid_Checker&action=edit&redlink=1) | self diagnostic, information and 24/7 online doctor | Android, Web-based / Web-site Widget |
| 16 | [Ghana](https://en.wikipedia.org/wiki/Ghana) | GH COVID-19 Tracker App |  | Android, iOS: *awaiting app store approvals* |
| 17 | [Hong Kong](https://en.wikipedia.org/wiki/Hong_Kong) | Stay Home Safe | quarantine enforcement | Unknown |
| 18 | [Hungary](https://en.wikipedia.org/wiki/Hungary) | VírusRadar | contact tracing | Android, iOS |
| 19 | [Iceland](https://en.wikipedia.org/wiki/Iceland) | [Rakning C-19](https://en.wikipedia.org/w/index.php?title=Rakning_C-19&action=edit&redlink=1) | route tracking | Android, IOS |
| 20 | [India](https://en.wikipedia.org/wiki/India) | [Aarogya Setu](https://en.wikipedia.org/wiki/Aarogya_Setu) | contact tracing | Android, iOS |
| 21 |  | COVA Punjab | contact tracing | Android, iOS |
| 22 |  | COVID-19 Feedback | feedback | Android |
| 23 |  | COVID-19 Quarantine Monitor | contact tracing, geofencing | TBA |
| 24 |  | Corona Kavach | information | Android (discontinued) |
| 25 |  | [GoK Direct](https://en.wikipedia.org/w/index.php?title=GoK_Direct&action=edit&redlink=1) | information | Android, iOS |
| 26 |  | Mahakavach | contact tracing | Android |
| 27 |  | Quarantine Watch | contact tracing | Android |
| 28 |  | Test Yourself Goa | self diagnostic | Android |
| 29 |  | Trackcovid-19.org | self diagnostic, syndromic surveillance | Web |
| 30 |  | Test Yourself Puducherry | self diagnostic | Android |
| 31 | [Israel](https://en.wikipedia.org/wiki/Israel) | [Hamagen (Hebrew: המגן‎ "the shield")](https://en.wikipedia.org/wiki/Hebrew_language) | contact tracing | Android, iOS |
| 32 | [Italy](https://en.wikipedia.org/wiki/Italy) | Covid Community Alert | CovidApp for citizens (proximity tracing and exposure notification, optional GPS location sharing), CovidDoc for doctors (scan patient QR code, log patient health status), web dashboard for epidemiologists (set parameters that trigger notifications) | Android, iOS, web |
| 33 |  | diAry “Digital Arianna” | GPS location tracing, exposure notification, awareness raising | Android, iOS |
| 34 |  | Immuni | contact tracing | Android, iOS |
| 35 |  | SM-COVID-19 | Contact Tracing | Android, iOS |
| 36 | [Jordan](https://en.wikipedia.org/wiki/Jordan) | AMAN (أمان"Safety") | Exposure Detection | Android, iOS |
| 37 | [Latvia](https://en.wikipedia.org/wiki/Latvia) | Apturi Covid | Exposure Detection | Android, iOS |
| 38 | [Malaysia](https://en.wikipedia.org/wiki/Malaysia) | Gerak Malaysia | [contact tracing, border crossingregistration](https://en.wikipedia.org/wiki/Border_crossing) | Android, iOS |
| 39 |  | MySejahtera | information | Android, iOS |
| 40 |  | MyTrace | contact tracing | Android, iOS |
| 41 | [Morocco](https://en.wikipedia.org/wiki/Morocco) | Wiqaytna (وقايتنا "Our prevention") | contact tracing | Android, iOS |
| 42 | [Netherlands](https://en.wikipedia.org/wiki/Netherlands) | [PrivateTracer](https://en.wikipedia.org/w/index.php?title=PrivateTracer&action=edit&redlink=1) | contact tracing | Android, iOS |
| 43 | [New Zealand](https://en.wikipedia.org/wiki/New_Zealand) | [NZ COVID Tracer](https://en.wikipedia.org/wiki/NZ_COVID_Tracer) | Point-of-interest journal | Android, iOS |
| 44 | [North Macedonia](https://en.wikipedia.org/wiki/North_Macedonia) | StopKorona! | contact tracing | Android, iOS |
| 45 | [Norway](https://en.wikipedia.org/wiki/Norway) | Smittestopp | contact tracing, route tracking | Android, iOS |
| 46 | [Poland](https://en.wikipedia.org/wiki/Poland) | ProteGO | contact tracing | Android, iOS |
| 47 | [Russia](https://en.wikipedia.org/wiki/Russia) | "Social Monitoring" | contact tracing | *under development* |
| 48 |  | Contact Tracer | Digital Contact Tracing and Alerting | Android |
| 49 | [Saudi Arabia](https://en.wikipedia.org/wiki/Saudi_Arabia) | Corona Map | Self Diagnostic Information | Android, iOS, Web |
| 50 | [Singapore](https://en.wikipedia.org/wiki/Singapore) | [TraceTogether](https://en.wikipedia.org/wiki/TraceTogether) | contact tracing | Android, iOS |
| 51 | [South Korea](https://en.wikipedia.org/wiki/South_Korea) | [Corona 100m](https://en.wikipedia.org/w/index.php?title=Corona_100m&action=edit&redlink=1) | contact tracing | Android (no longer available) |
| 52 |  | Self-Diagnosis app | self-diagnostic | Android, iOS |
| 53 |  | Self-Quarantine app | isolation registration | Android, iOS |
| 54 | [South Africa](https://en.wikipedia.org/wiki/South_Africa) | [Covi-ID](https://en.wikipedia.org/w/index.php?title=Covi-ID&action=edit&redlink=1) | contact tracing, health credential management | Android, iOS, Web |
| 55 | [Sri Lanka](https://en.wikipedia.org/wiki/Sri_Lanka) | Self Shield (Formerly COVID Shield) | Self-Health Checking and monitoring, AI driven breathing performance assessment, Quarantine Monitoring and Support, reporting test state, demographic mapping | Android |
| 56 | [Switzerland](https://en.wikipedia.org/wiki/Switzerland) | [SwissCovid](https://en.wikipedia.org/w/index.php?title=SwissCovid&action=edit&redlink=1) | contact tracing | Android, iOS |
| 57 | [United Kingdom](https://en.wikipedia.org/wiki/United_Kingdom) | [COVID Symptom Study, formerly Covid Symptom Tracker](https://en.wikipedia.org/wiki/COVID_Symptom_Study) | self-diagnostic | Android, iOS |
| 58 |  | [NHS COVID-19](https://en.wikipedia.org/wiki/NHS_COVID-19) | multipurpose | Android, iOS |
| 59 | [United States](https://en.wikipedia.org/wiki/United_States) | [COVID-19 Screening Tool](https://www.apple.com/covid19) | self-diagnostic | Web |
| 60 |  | CovidSafe | self-diagnostic, contact tracing | Android, iOS |
| 61 |  | How We Feel | self-diagnostic | Android, iOS |
| 62 |  | Private Kit: Safe Paths | contact tracing | Android, iOS |
| 63 |  | [Covid Watch](https://en.wikipedia.org/w/index.php?title=Covid_Watch&action=edit&redlink=1) | exposure alerts | Android, iOS |
| 64 |  | coEpi | self-reporting | Android, iOS |
| 65 |  | NOVID | contact tracing | Android, iOS |
| 66 | [Việt Nam](https://en.wikipedia.org/wiki/Vietnam) | NCOVI | medical reporting | Android, iOS |
| 67 | global | World Health Organization COVID-19 App | information | Android, iOS |
| 68 | global | Coalition App | contact tracing | Android, iOS, third party hardware |
